# Update on SLC6A14 in lung and gastrointestinal physiology and physiopathology: focus on cystic fibrosis

**DOI:** 10.1007/s00018-020-03487-x

**Published:** 2020-03-12

**Authors:** Manon Ruffin, Julia Mercier, Claire Calmel, Julie Mésinèle, Jeanne Bigot, Erika N. Sutanto, Anthony Kicic, Harriet Corvol, Loic Guillot

**Affiliations:** 1 Sorbonne Université, INSERM UMR S 938, Centre de Recherche Saint‑Antoine (CRSA), Paris, France; 2 Telethon Kids Institute, University of Western Australia, Nedlands, WA Australia; 3 School of Public Health, Curtin University, Bentley, WA Australia; 4 Centre for Cell Therapy and Regenerative Medicine, Medical School, The University of Western Australia, Nedlands, WA Australia; 5 Department of Respiratory and Sleep Medicine, Perth Children’s Hospital, Nedlands, WA Australia; 6 Pneumologie Pédiatrique, APHP, Hôpital Trousseau, Paris, France

**Keywords:** ATB^0,+^, Amino acid transporter, CFTR, Nitric oxide, Pulmonary, Intestine

## Abstract

The solute carrier family 6 member 14 (SLC6A14) protein imports and concentrates all neutral amino acids as well as the two cationic acids lysine and arginine into the cytoplasm of different cell types. Primarily described as involved in several cancer and colonic diseases physiopathological mechanisms, the *SLC6A14* gene has been more recently identified as a genetic modifier of cystic fibrosis (CF) disease severity. It was indeed shown to have a pleiotropic effect, modulating meconium ileus occurrence, lung disease severity, and precocity of *P. aeruginosa* airway infection. The biological mechanisms explaining the impact of SLC6A14 on intestinal and lung phenotypes of CF patients are starting to be elucidated. This review focuses on SLC6A14 in lung and gastrointestinal physiology and physiopathology, especially its involvement in the pathophysiology of CF disease.

## Introduction

Cystic fibrosis (CF), the most common lethal autosomal recessive genetic disease in Caucasians, is caused by variants in the gene encoding the cystic fibrosis transmembrane conductance regulator (*CFTR*), a chloride channel expressed ubiquitously within epithelia [1–3].

Symptoms can occur as early as birth with meconium ileus (MI), a severe neonatal intestinal obstruction affecting around 15% of CF neonates. This is followed by manifestations of the disease in other organs such as the liver, the pancreas, and the intestine, with lung complications as the main cause of morbidity and mortality in CF patients. In the lungs, absence or dysfunction of CFTR proteins results in altered salt and water transport through the airway epithelium leading to an altered mucociliary clearance, progressive colonization with different pathogens, exacerbation of inflammation, and lung tissue damage. Among the multitude of pathogens colonizing the CF lungs, *Pseudomonas aeruginosa* is the most common and life-threatening pathogen. Indeed, *P. aeruginosa* chronic lung colonization has been associated with a more severe lung disease and reduced survival [4].

Although CF is a monogenic disease, considerable phenotypic diversity is observed in patients carrying identical *CFTR* variants [5–7]. In addition to environmental factors, twins and siblings’ studies have revealed that genetic modifiers outside the *CFTR* locus are involved in this interindividual variability [5]. It is expected that these modifier genes account for 50% of the lung function variation. The current challenge is to identify these variants and determine how they contribute to the severity of the disease by performing in vitro/in vivo functional studies. Among the several modifier genes identified thus far, the solute carrier family 6 member 14 (*SLC6A14*, also known as *ATB*^*0,*+^) has been shown to have pleiotropic effect in CF [8–14]. It was first identified as a modifier of MI occurrence [14], and then associated with lung disease and age at first *P. aeruginosa* infection [8, 10]. SLC6A14, the protein encoded by this gene, belongs to the solute carrier family 6 and uses the energy provided by Na^+^ and Cl^−^ gradients to import and concentrate all neutral amino acids as well as the two cationic acids lysine and arginine into the cytoplasm of different cell types. Besides the genotype/phenotype associations, the biological mechanisms explaining the impact of SLC6A14 on intestinal and lung phenotypes of CF patients are beginning to be elucidated as evidenced by recent studies [15–17]. This review focuses on SLC6A14 in the context of CF, especially its involvement in the pathophysiology of CF lung and gastrointestinal disease.

## SLC6A14 expression and regulation in the lung and gastrointestinal tract

*SLC6A14* gene is located on chromosome X and was cloned in 1999 from a mammary gland cDNA library [18]. This gene produces two transcripts (ENST00000598581.3 and ENST00000463626.1) but only one codes for a protein (ENSG00000087916.7) which is comprised of 642 amino acids with an expected molecular weight of 72 kDa. SLC6A14 is a plasma membrane protein belonging to the solute carrier 6 (SLC6) family which contains 21 human proteins based on the similarity in their amino acid sequences [19]. Although no structural studies have been conducted on SLC6A14 specifically, crystal structure and structural studies on others members of the SLC6 family, as well as topological domain analysis (https://uniprot.org/uniprot/Q9UN76) suggest that SLC6A14 N- and C-terminal domains are cytoplasmic and that the sequence includes 12 putative transmembrane domains and 1 large extracellular domain between transmembrane domains 3 and 4 [19]. In the endoplasmic reticulum, the quality control of SLC6A14 folding involves interactions with the heat shock proteins HSP70 and HSP90 [20]. SLC6A14 trafficking from the endoplasmic reticulum to the Golgi apparatus depends on its interaction with the cargo-recognizing protein SEC24 isoform C and the coatomer II (COPII) complex [21]. Further studies are needed to fully understand the mechanisms allowing SLC6A14 trafficking to the plasma membrane.

*SLC6A14* mRNA was initially shown to be expressed mainly in the lung, fetal lung, trachea, and salivary gland [18]. Microarray and RNA-sequencing data obtained from Expression Atlas public resource confirm that *SLC6A14* is predominantly expressed in human and mouse lung tissue (Table 1). However, *SLC6A14* is also detected albeit in extremely low levels in gastrointestinal tissues including intestine and colon.

**Table 1:**
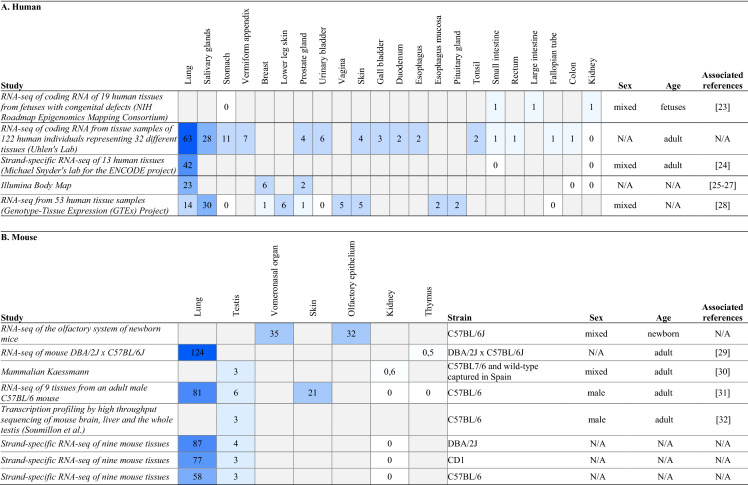
*SLC6A14* transcript expression in human (A) and mouse (B) ( Modified from: Expression Atlas; https://www.ebi.ac.uk)

Numbers refer as transcripts per million, the gradation of blue illustrates the abundance of the transcript *SLC6A14*

### SLC6A14 expression and regulation in the lung

Before SLC6A14 mRNA and protein were identified in the human lung, Galietta et al. demonstrated the presence of a Na^+^-dependent amino acid transport at the apical membrane of bronchial epithelial cells isolated from CF or non-CF subjects [32]. Upon showing that *SLC6A14* mRNA was strongly expressed in the human lung and trachea compared to other organs [18], Sloan et al. also showed that SLC6A14 protein was expressed in membrane fractions of human airway and distal lung samples from normal, emphysema, and CF patients [33]. Interestingly, the authors pointed out differences in the molecular mass of SLC6A14 protein detected either in the airways or in the distal lung, which was due to alternative splicing or posttranslational modification. Several studies have also shown expression of SLC6A14 in various cell lines of either airway or distal epithelial cell origin, as well as in primary bronchial epithelial cells (Table 2). In the alveolar A549 cell line, SLC6A14 protein was either detected or absent according to the study considered [34, 35]. Di Paola et al. observed that primary cells from individuals with CF or healthy donors showed a similar mRNA level for SLC6A14 [17], suggesting that *CFTR* pathogenic variants had no impact on *SLC6A14* mRNA expression. Interestingly, a recent study using single RNA sequencing revealed that *SLC6A14* expression was reduced in alveolar type II cells from idiopathic pulmonary fibrosis (IPF) patients compared to controls [36]. Via single-cell analysis, the authors also showed that *SLC6A14* was expressed in basal, club cells, and alveolar type 2 cells.

**Table 2:** Expression of SLC6A14 at the mRNA and protein levels in the human respiratory tract

Sample	Tissue/cell types	mRNA	Protein	Ref
Tissues	Lung tissue samples		Expressed (WB)	[33]
Cell lines	Calu-3	Expressed (qPCR)	–	[37]
	NCI-H69	Expressed (qPCR)	–	[38]
	A549, BEAS-2B	Barely detectable (qPCR)	Undetected (WB)	[34]
	Calu-3, NCI-H441	Expressed (qPCR)	Expressed (WB)	[34]
	Calu-3, CFBE41o-	Expressed (qPCR)	–	[17]
	A549	–	Expressed (WB)	[35]
Primary cells	Alveolar type 2 cells isolated from control and idiopathic pulmonary fibrosis (IPF) lung tissue	Expressed. Reduced in IPF cells (scRNAseq, qPCR)	–	[36]
	Bronchial epithelial cells isolated from posttransplant tissue from healthy donors and CF patients	Expressed (qPCR)	–	[17]

*Calu-3* human lung adenocarcinoma cell line, *NCI-H69 and A549* human lung carcinoma cell line, *BEAS-2B* human bronchial epithelial cell line from a normal subject, *NCI-H441* human lung papillary adenocarcinoma cell line, *CFBE41o-* human cystic fibrosis bronchial epithelial cell line, – not studied, *qPCR* quantitative polymerase chain reaction, *WB* Western blot

In vitro, Gorrieri et al. observed that *SLC6A14* transcripts were enhanced in bronchial epithelial cells exposed to interleukin-4 [39]. In the human glandular bronchial epithelial cell line Calu-3 and in primary human bronchial epithelial cells, flagellin or lipopolysaccharide from *P. aeruginosa* exposures increase the expression of *SLC6A14* mRNA [17]. Finally, in relation to CF, a study using HEK-293 cells over-expressing SLC6A14 showed that SLC6A14 protein expression was reduced by suprapharmacological concentrations of Vx-770, a CFTR potentiator [40].

### SLC6A14 expression and regulation in the gastrointestinal tract

In the human gastrointestinal tract, SLC6A14 appears to be heterogeneously expressed (Table 3). Sloan et al. first detected *SLC6A14* mRNA in the stomach, although its levels were significantly lower than those observed in lung samples [18]. Two studies then detected *SLC6A14* transcripts in mucosal biopsies from duodenum and rectum, respectively [41, 42]. Finally, Anderson et al. compared the expression of *SLC6A14* mRNA throughout the gastrointestinal tract and showed that stomach, duodenum, and descending colon expressed high levels of *SLC6A14* transcripts, while low levels were found in jejunum, ileum, ascending colon, and transverse colon [43]. Conflicting results have been obtained on the expression of SLC6A14 in the human colon epithelial cell line Caco-2, reporting either some or no SLC6A14 transcripts or protein [16, 42–44]. Interestingly, in mice, *SLC6A14* mRNA expression is negligible in the ileum of control animals, but is strongly induced in epithelial ileal cells of CF mice [45].

**Table 3:** Expression of SLC6A14 at the mRNA and protein level in human gastrointestinal tract

Sample	Tissues/cell types	mRNA	Protein	Ref
Tissues	Intestinal epithelium (cholera patients)	Expressed	Expressed (IHC)	[42]
	Gastrointestinal tissues	Expressed	–	[43, 46]
Cell lines	Caco-2	Expressed	–	[42]
		Undetectable	–	[43]
		–	Expressed (WB & IF)	[44]
		Not expressed (PCR)	Not expressed (WB)	[16]
	CCD841, HCT116, HT29, LS174T	–	Expressed (WB)	[44]

*Caco-2, HT29 and LS174T* human adenocarcinoma colorectal epithelial cell lines, *CCD841* normal human colon epithelial cell line, *HCT116* human colon epithelial cell line from colorectal carcinoma, *HT29* *and* *LS174T* human epithelial cell lines from colon adenocarcinoma (– not studied, *WB* Western blot, *IF* immunofluorescence, *IHC* immunohistochemistry)

In vitro, it has been showed that SLC6A14 expression may be modulated by several factors including toxins, bacterial constituents, and proinflammatory cytokines. Indeed, Flach et al. showed that *SLC6A14* mRNA levels are significantly increased after 18 h of stimulation with cholera toxin in Caco-2 cells [42]. Other molecules have also been shown to regulate SLC6A14 expression. For example, in porcine intestinal cells, Wang et al. showed that *SLC6A14* mRNA was increased by L-tryptophan [47]. Ikpa et al. also showed that antibiotic treatment of CF mice induces an important reduction of *SLC6A14* transcripts in ileal epithelial cells [45].

## Genetic association studies in CF

**Fig. 1:**
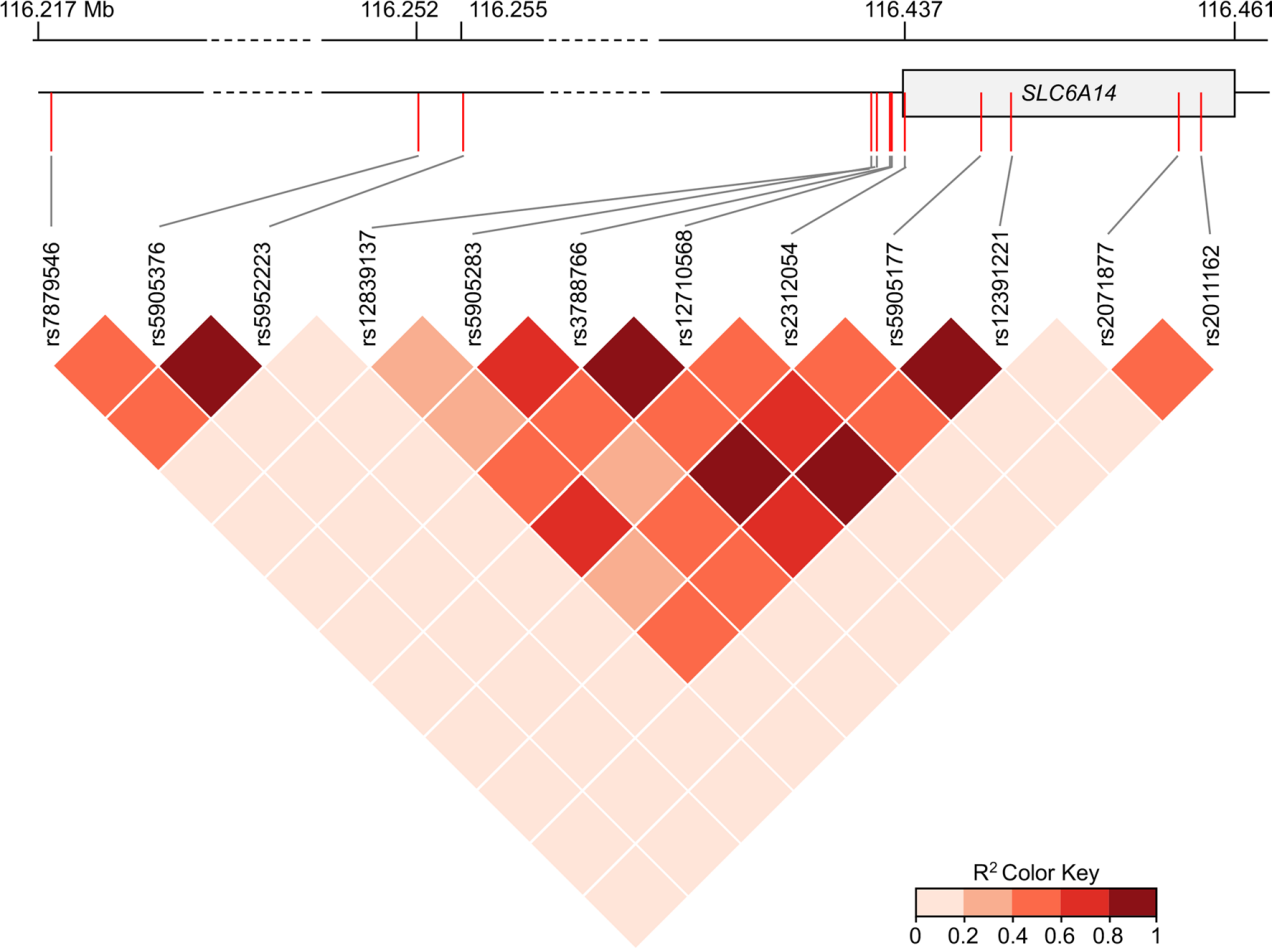
Linkage disequilibrium (LD) pattern of the twelve *SLC6A14* genetic variants studied. The dark red squares indicate pairs in strong LD. *R*^2^ are from https://ldlink.nci.nih.gov

Given the diversity of phenotypic severity in CF patients with the same causal *CFTR* variants, several genetic studies have been conducted to identify CF modifier genes. Among the identified loci, one locus on chromosome X, near the *SLC6A14* gene, was associated with a variability in the severity of CF clinical manifestations including lung disease severity/pulmonary infections or presence of MI/onset of digestive symptoms (Table 4). Linkage disequilibrium pattern of the different genetic variants studies in this review is shown in Fig. 1.

**Table 4:** Genetic associations tested between *SLC6A14* variants and digestive and pulmonary manifestations in CF patients

rs ID (Alleles)	MAF	Variant localization	Association with	Number of CF patients	Cohort/patients characteristics	Ref
rs7879546 (T/C)	0.41	Intergenic	Lung disease severity	6365	Mean age: 19.5 years; PI (99.8%); F508del homozygotes (65%)	[8]
rs5905376 (C/A)	0.23	Intergenic	Lung disease severity			
rs5952223 (C/T)	0.23	Intergenic	Lung disease severity			
rs12839137 (G/A)	0.12	Intergenic	Presence of meconium ileus	6135	Two independent cohorts (patients with two severe *CFTR* mutations): 3,763 North American (F508del homozygotes 71.4%) and 2,372 French (> 6 years old) and American patients	[14]
			No association with pediatric lung disease severity	815	Mean age of lung function measurements: 12.63 years; F508del homozygotes: 62.3%	[10]
			No association with age of first infection by *P. aeruginosa*	730	Median age at first detection of positive *P. aeruginosa* culture: 5.55 years; F508del homozygotes: 61%	
			No association with early exocrine pancreatic disease	126	Median age of the first available IRT measurement: 0.36 years; F508del homozygotes: 60.3%	
			No association with early exocrine pancreatic damage	111	Patients from Colorado, median age at IRT measurement: 2 days; F508del homozygotes: 56%	[11]
rs5905283 (A/C)	0.47	Intergenic (2 KB Upstream Variant)	Presence of meconium ileus	6,135	Two independent cohorts (patients with two severe *CFTR* mutations): 3,763 North American (F508del homozygotes 71.4%) and 2,372 French (> 6 years old) and American patients	[14]
			Pediatric lung disease severity	815	Mean age of lung function measurements: 12.63 years; F508del homozygotes: 62.3%	[10]
			No association with age of first infection by *P. aeruginosa*	730	Median age at first detection of positive *P. aeruginosa* culture: 5.55 years; F508del homozygotes: 61%	
			No association with early exocrine pancreatic phenotypes	126	Median age of the first available IRT measurement: 0.36 years; F508del homozygotes: 60.3%	
			No association with early exocrine pancreatic damage	111	Patients from Colorado, median age at IRT measurement: 2 days; F508del homozygotes: 56%	[11]
rs3788766 (G/A)	0.36	Regulatory region	Presence of meconium ileus	6,135	Two independent cohorts (patients with two severe *CFTR* mutations): 3,763 North American (F508del homozygotes 71.4%) and 2,372 French (> 6 years old) and American patients	[14]
			Pediatric lung disease severity	815	Mean age of lung function measurements: 12.63 years; F508del homozygotes: 62.3%	[10]
			Age of first infection by *P. aeruginosa*	730	Median age at first detection of positive *P. aeruginosa* culture: 5.55 years; F508del homozygotes: 61%	
			No association with early exocrine pancreatic phenotypes	126	Median age of the first available IRT measurement: 0.36 years; F508del homozygotes: 60.3%	
			No association with early exocrine pancreatic damage	111	Patients from Colorado, median age at IRT measurement: 2 days; F508del homozygotes: 56%	[11]
			Early pulmonary symptoms	79	Brazilian patients	[12]
			*P. aeruginosa* infection	83		
			Presence of meconium ileus	6,770	Patients with two severe *CFTR* mutations associated with PI; F508del homozygotes: 64.2%	[9]
rs12710568 (G/C)	0.31	Regulatory region	Presence of meconium ileus	6,770	Patients with two severe *CFTR* mutations associated with PI; F508del homozygotes: 64.2%	[9]
rs5905177 (C/T)	0.35	*SLC6A14* intron	Presence of meconium ileus			

Chromosomic position (forward strand); minor allele in the European population, Minor allele Frequency (MAF), data were collected from Ensembl, 1000 Genomes, European population. *IRT* immunoreactive trypsinogen, *PI* pancreatic insufficiency

The initial evidence showing that *SLC6A14* may be a modifier gene in CF has been described by Sun et al. in a “genome wide association study” (GWAS) involving 6135 CF patients [14]. This study identified a significant association between susceptibility to MI and three *SLC6A14* genetic variants (rs12839137, rs5905283, and rs3788766). In a study involving more than 6700 CF patients from the International CF Gene Modifier Consortium, Gong et al. recently replicated the association between susceptibility to MI and rs3788766 [9]. This study also identified an association between MI susceptibility and two new variants (rs12710568 and rs5905177) located within the *SLC6A14* regulatory region and *SLC6A14* intron, respectively. Several groups performed sex-specific association analysis based on the fact that *SLC6A14* gene is located within the region associated with random X-inactivation [9, 14]. Interestingly, they found higher odd ratios in male than in female only for genetic variants associated with susceptibility to MI.

Li et al*.* further assessed the association of MI risk alleles of *SLC6A14* with other CF co-morbidities, such as the lung disease severity and age at first *P. aeruginosa* infection [10]. Their study involved 815 CF Canadian pediatric patients who were genotyped for the following *SLC6A14* variants: rs12839137, rs5905283, and rs3788766. Among the variants studied, rs5905283 and rs3788766 risk alleles were associated with pediatric lung disease severity; whilst rs3788766 variant was associated with age at first *P. aeruginosa* infection, as confirmed later in a smaller cohort [12]. In 2015, a GWAS involving 6,365 patients confirmed that *SLC6A14* modifies the severity of the lung disease in CF [8]. Indeed, the authors found a significant association between genotypes of three *SLC6A14* intergenic variants (rs7879546, rs5905376, and rs5952223) and the lung disease severity.

Gong et al. recently integrated GWAS and tissue-specific gene expression data to determine whether modifier loci on chromosome X (encompassing *SLC6A14*) influence *SLC6A14* mRNA expression levels in different tissues [9]. This kind of analysis indicates whether eQTL (expression quantitative trait loci) colocalize with loci associated with CF phenotypes that may indicate the existence of a genetic regulator. Their results showed that *SLC6A14* mRNA expression in CF nasal epithelia and in the pancreas colocalize with the lung disease and MI-associated variants, respectively, suggesting that each locus impacts *SLC6A14* expression with tissue specificity. Besides, neither association between *SLC6A14* genetic variants and early exocrine pancreatic phenotype nor immunoreactive trypsin levels at birth have been found [10, 11].

## Putative biological roles of SLC6A14 in CF

Several studies showed that SLC6A14 plays a primary role as an amino acid transporter in various epithelial cells and models [32, 48–52]. Taken together, SLC6A14 expression data in human and genetic studies suggest that SLC6A14 may have an important role in the lung and intestinal pathophysiology of CF patients (see Parts 1 and 2).

### SLC6A14 function in the lung

**Fig. 2:**
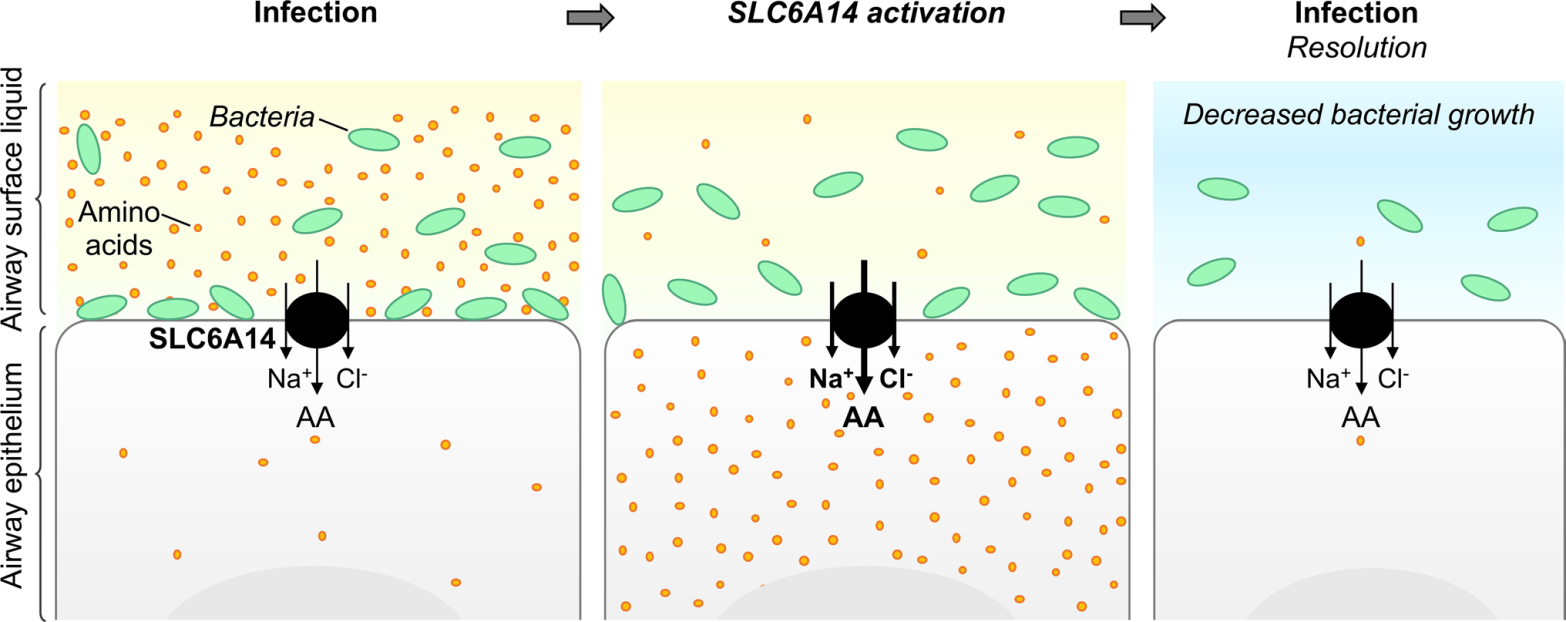
Proposed mechanism of the role of SLC6A14 in the host response against *P. aeruginosa*. *AA* amino acids, *Na*^*+*^ sodium ions, *Cl*^*−*^ chloride ions

In the lung, it was first suggested that the apical transport of amino acid in the airway epithelial cells may play an important role in infection resolution [32] as pathogens need amino acids to proliferate into the airways. The authors suggested that amino acid transporter at the apical membrane may be activated following infection to rapidly decrease the amino acid concentration of the airway surface liquid (ASL) (Fig. 2). This phenomenon could then help to fight against infections. This hypothesis is supported by the unique characteristics of SLC6A14 allowing it to strongly concentrate all essential amino acids into the cytoplasm of epithelial cells.

As *SLC6A14* genetic variants have been associated with age at first acquisition of *P. aeruginosa* in CF patients, Di Paola et al. sought to determine how SLC6A14 might impact the airways colonization by these bacteria [17]. They suggested that exposure to *P. aeruginosa* increased *SLC6A14* mRNA expression, inducing a decrease in amino acid concentration in the ASL that resulted in a decrease in *P. aeruginosa* attachment to the airway epithelial cells rather than a reduced viability of planktonic *P. aeruginosa*. Indeed, they found that purified flagellin from *P. aeruginosa* enhanced *SLC6A14* mRNA expression and SLC6A14-dependent arginine import in Calu-3 cells and in primary airway epithelial cells from non-CF and CF patients. Moreover, they showed that pharmacological inhibition of SLC6A14 increased *P. aeruginosa* attachment in non-CF primary airway epithelial cells and slightly in the bronchial epithelial cell line CFBE41o-.

**Fig. 3:**
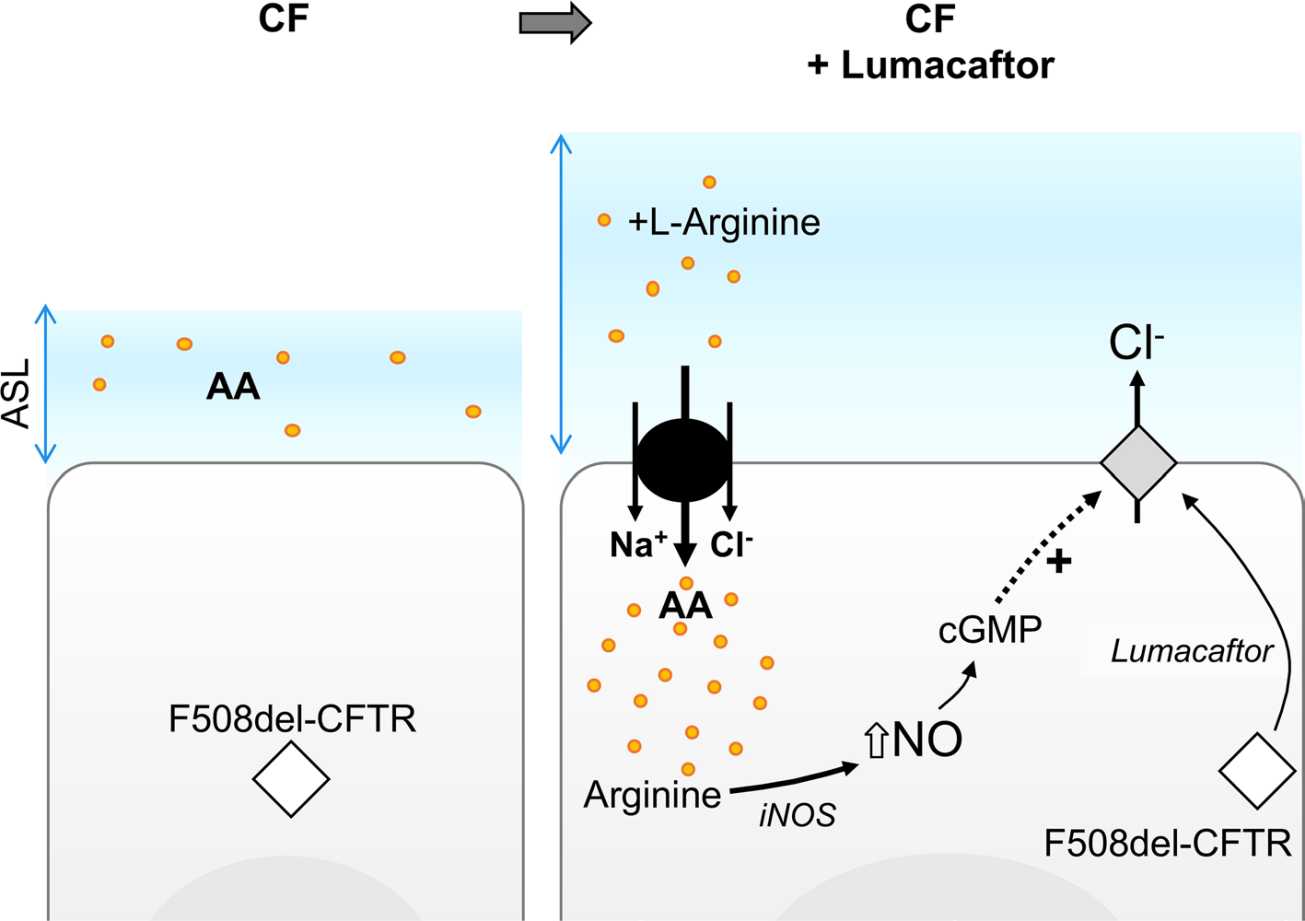
Relationships between SLC6A14, F508del-CFTR, and nitric oxide signaling pathway. *CF* cystic fibrosis, *ASL* airway surface liquid, *AA* amino acids, *Na*^*+*^ sodium ions, *Cl*^*−*^ chloride ions, *HCO3-* bicarbonate ions, *iNOS* inducible NO synthetase, *NO* nitric oxide

Ahmadi et al. recently reported that arginine transport through SLC6A14 increased F508del-CFTR Cl^−^ efflux in CF airway epithelial cells stimulated with or without a CFTR corrector, lumacaftor [15]. They also observed that this increase in CFTR function induced an increase in the ASL height and that the potentiation of F508del-CFTR channel function in CF cells induced by SLC6A14 arginine uptake occurred via the nitric oxide (NO) signaling pathway (Fig. 3). Finally, they suggested that SLC6A14 activation may be considered as a complement therapy to CFTR correction and potentiation in CF patients.

### SLC6A14 function in the gastrointestinal tract

Ahmadi et al. also conducted another study to determine the biological function of SLC6A14 in the murine gastrointestinal tract [16]. They first demonstrated that SLC6A14 is a major apical amino acid transporter in the murine colon. Indeed, *Slc6a14*(-/y) mice exhibited almost 75% reduction in apical arginine transport compared to WT mice. In CF mice, they observed that *Slc6a14* disruption induced a reduction in weight gain and BMI post-weaning and worsen the intestinal phenotype, i.e., decreased F508del-CFTR-mediated fluid secretion. They also highlighted that SLC6A14 does not seem to affect the processing or stability of F508del-CFTR neither co-immunoprecipitated with F508del-CFTR in an F508del-CFTR BHK over-expression system, which led them to investigate intracellular signaling such as NO synthesis. They observed that SLC6A14 inhibition impaired arginine uptake by intestinal epithelial cells inducing both a decrease in NO production and cGMP regulation of F508del-CFTR. These results suggest that an increase in SLC6A14 activity may enhance NO production and F508del-CFTR residual activity in CF tissues. However, it is not known whether these mechanisms are conserved in humans.

It has to be emphasized that in the lung or the intestine, functional studies were conducted with either over-expression (plasmid) or inhibition experiments (siRNA) of the whole gene. The role of the specific genetic variants identified in genetic studies (see Part entitled "Genetic association studies in CF") is not yet known and further elucidation is warranted.

## *SLC6A14* in non-CF diseases

### SLC6A14 expression in other diseases

SLC6A14 expression has been shown to be differentially up-regulated in several pathological contexts (Table 5), especially in cancer and colonic diseases (reviewed in [53]).

**Table 5:** Expression of SLC6A14 in other diseases

Disease		SLC6A14 expression	Methods	Ref
Cancer	Cervical	Up-regulated	PCR, IF/IH, HIS	[54]
	Colorectal	Up-regulated	PCR, Northern blot, IH	[55]
	Pancreatic	Up-regulated	Microarray, qPCR, WB, IF, IH	[56, 57]
	Breast (ER +)	Up-regulated	PCR, IF	[58]
Colonic diseases	Crohn’s disease	Up-regulated	qPCR	[46]
	Ulcerative colitis	Up-regulated	qPCR, microarray	[41, 59, 60]
	Long vs. short duration of ulcerative colitis	Down-regulated	Microarray	[61]
	Ischemic or infectious colitis	Up-regulated	qPCR	[60]
Cholera	Acute vs. convalescence phase	Up-regulated	Microarray, qPCR, IH	[42]
IPF	IPF	Down-regulated	scRNAseq	[36]
	IPF vs. NSIP	Up-regulated	Microarray	[62]

*ER+* estrogen receptor-positive, *IPF* idiopathic pulmonary fibrosis, *NSIP* non-specific interstitial pneumonia. If not specifically mentioned, the expression of SLC6A14 is relative to control patients. *IF* immunofluorescence, *IH* immunohistochemistry, *HIS* hybridization in situ, *PCR* polymerase chain reaction, *WB* western blot

#### Cancer

SLC6A14 is significantly upregulated in tissues from cervical [54], colorectal [55], pancreatic [56, 57], and estrogen receptor-positive (ER+) breast cancer [58]. In ER+ breast cancer, high expression of *SLC6A14* mRNA has been correlated with a better survival among patients [63]. Using a mouse model of spontaneous breast cancer, Babu et al. showed that its development and progression was significantly decreased when the mice were crossed with *Slc6a14*^*−/−*^ mice [64]. The molecular mechanisms associated with these up- or down-regulations are largely unknown. SLC6A14 expression was shown to be regulated by estrogen [58] which explains its specific increased expression in ER+ but not in ER- breast cancer. Also, inverse expression patterns of *SLC6A14* mRNA and the microRNA (miR)-23a [65, 66] were found suggesting its regulatory effect. However, no functional studies (using miR mimic or inhibitors) confirmed miR-23a involvement in *SLC6A14* expression regulation. In contrast, inverse correlation of miR-23b-3p [67] and *SLC6A14* expression was recently confirmed. Functional studies have shown that the downregulation of SLC6A14 observed in endocrine therapy (ER + breast cancer standard of care)-resistant cells is associated with an increase of miR-23b-3p [63].

#### Colonic diseases

*SLC6A14* mRNA levels are significantly higher in colonic mucosal specimens obtained from patients with Crohn’s disease compared to controls [46]. *SLC6A14* expression was also increased in rectal and colonic biopsies from patients with ulcerative colitis or infectious/ischemic colitis compared to controls, suggesting that *SLC6A14* upregulation might be the result of the inflammatory context rather than a specific pathophysiological consequence of the ulcerative colitis [41, 59, 60, 68]. Low et al. further observed that *SLC6A14* was down-regulated in colonic biopsies from patients with long-duration of ulcerative colitis compared with patients with short duration [61]. In rats, D’Argenio et al. showed that experimental colitis induced a marked decrease in *SLC6A14* transcript expression in the colon [69]. Finally, Kou et al. found that colon cancer cell lines overexpressed SLC6A14 compared to normal colon cells [44].

#### Idiopathic pulmonary fibrosis

*SLC6A14* has been found to be downregulated in alveolar-type II cells of idiopathic pulmonary fibrosis (IPF) patients [36], while it is overexpressed in specimens from explanted lungs of patients with non-specific interstitial pneumonia compared to specimens from IPF patients [62].

#### Infectious diseases

*SLC6A14* mRNA levels have been shown to be increased in biopsies of duodenum collected during the acute phase of cholera compared to biopsies collected during convalescence phase [42].

### Genetic associations studies in other diseases

Four additional *SLC6A14* genetic variants have been associated with phenotypic variability in other diseases than CF (Table 6).

**Table 6:** Genetic associations tested between *SLC6A14* variants and diseases

rs ID (alleles)	MAF	Variant localization	Association with	Number of patients/cohort characteristics	Ref
rs2312054 (A/T)	0.21	*SLC6A14* intron	Food intake	344 children, age 7–8 years	[70]
rs12391221 (C/A)	0.30	*SLC6A14* intron	Food intake	344 children, age 7–8 years	[70]
rs2071877 (C/T)	0.30	*SLC6A14* intron	Obesity	1267 obese adults and 649 lean controls (French)	[71]
			Adiposity	344 children, age 7–8 years	[70]
rs2011162 (C/G)	0.45	*SLC6A14* exon 14; 3′UTR	Obesity	Two independent cohorts: 117 obese and 182 controls (Finnish); 837 obese and 968 controls (Finnish and Swedish)	[72]
			Obesity	1267 obese adults and 649 lean controls (French)	[71]
			Reduced fat oxidation in women	722 obese subjects of white European origin (541 women, 181 men), age 20–25 years	[73]
			Male infertility	370 infertile men and 241 fertile controls (Macedonian and Slovenian)	[74]
rs2312054(A)/ rs2071877(C)/ rs2011162(G) haplotype			Male infertility	370 infertile men and 241 fertile controls (Macedonian and Slovenian)	[74]

Chromosomic position (forward strand); Alleles (Minor), Minor allele Frequency (MAF), data were collected from Ensembl, 1000 Genomes, European population

*SLC6A14* genetic variants have been associated with obesity in different populations (Table 6). In a candidate gene analysis, later replicated in an independent cohort, Suviolahti et al. found significant differences in *SLC6A14* rs2011162 genetic variant allele frequencies between obese and non-obese subjects [72]. Another study suggested an association between the rs2011162 and fat oxidation in women which may be, when not adapted to fat intake, responsible to weight gain over time [73]. Finally, in a French family cohort study comprising of 1,267 obese adults and 649 lean control subjects, Durand et al. found a significant association between rs2011162 genetic variant and obesity. They observed that the risk allele was associated with higher body fat and modified perception of hunger and satiety in adult women with moderate obesity and in obese girls [71]. Durand et al. also identified an association between *SLC6A14* rs2071877 genetic variant and obesity in a French cohort [71]. This variant has also been associated with sum of triceps and subscapular skinfolds thickness, an objective measure of adiposity, in boys 7–8 of age [70]. Finally, Miranda et al. also found evidences of associations between two other genetic variants, rs2312054 and rs12391221, and several parameters used to assess the food intake in children [70].

*SLC6A14* genetic variants have been also associated with male infertility. Indeed, Noveski et al. found that rs2011162 alone and rs2011162(G)/rs2071877(C)/rs2312054(A) haplotype were differently distributed among fertile and infertile groups in their cohort [74]. As rs2011162 is located within the 3′UTR region of *SLC6A14*, they investigated the possible consequences of this genetic variation on the RNA secondary structure. They found a significant structural effect of this genetic variant that may result in a differential mRNA expression depending on the allele.

It was recently shown that SLC6A14 expression quantitative trait loci (eQTL) from nasal epithelial cells and pancreas tissues coincide with lung disease and meconium ileus-associated variants, respectively, supporting an important role for *SLC6A14* variants in CF [9]. However, whether in CF or other diseases, the functional in vitro/in vivo consequences of identified *SLC6A14* genetic variants on SLC6A14 protein expression and/or function have never been studied. This gap in knowledge needs to be addressed to better understand the molecular mechanisms by which SLC6A14 affect phenotypes or diseases. *SLC6A14* genetic variants described in this review are located in the non-coding region either intergenic or located in the regulatory region (promoter), introns, or in the 3′UTR region of *SLC6A14,* and, subsequently, do not modify the amino acid sequence of SLC6A14 protein. However, these variants may have multiple effects not only on SLC6A14, but also on nearby and/or distant genes. For example, genetic variants located in the promoter may affect transcriptional activity by altering transcription factor binding. Other functional consequences of these SNPs have to be studied including DNA methylation and histone modifications, alternative splicing, conformation and stability of mRNA as well as structure, expression level, and function of proteins. Even if bioinformatic tools may predict functional consequences of genetic variants, downstream in vitro/in vivo experimental studies will also be necessary. Successfully used for several complex traits, genome-editing technologies may also be used to create isogenic cell lines with specific alleles to assess their functionality including chromatin structure, transcription factor binding, gene and protein expression, and specific cellular assays [75]. Furthermore, as previously mentioned, in addition to the impact of genetic variants, SLC6A14 expression can be regulated by environmental factors (inflammatory molecules, pathogens, and pharmacological treatments) and possibly age (fetal vs. adult, Table 1). Thus, the development of different experimental models to identify the causality of SLC6A14 expression and function variability over the course of disease progression will be a major challenge.

## Conclusion

SLC6A14 seems to be predominantly expressed in epithelial cells of the human lung and to a lesser extent in the human gastrointestinal epithelium. Importantly, SLC6A14 expression profiles appear to be different between human and mice, suggesting that studies investigating the biological roles of this protein in murine models may not directly correlate with findings obtained in human models. Several groups have observed that SLC6A14 levels are up- or down-regulated in pathological conditions, however, the mechanisms involved in these dysregulations have mostly not been elucidated. Moreover, some studies have demonstrated that inflammatory mediators and pathogen molecules may impact SLC6A14 expression.

Furthermore, genetic studies highlight that *SLC6A14* genetic variants modulate the severity of digestive and pulmonary diseases in CF patients. The biological function of this pleiotropic modifier gene is not fully explained and the biological direct consequences of identified variants in genetic studies remained to be clarified. However, some recent studies suggest that SLC6A14 may play an important role in the response to respiratory infection and fluid secretion related to CFTR. Thus, SLC6A14 may be a potential therapeutic target to improve anti-infective response and CFTR function and/or correction in CF patients in a personalized way.
